# Classification-Based Approaches to Myopia Control in a Taiwanese Cohort

**DOI:** 10.3389/fmed.2022.879210

**Published:** 2022-06-10

**Authors:** Meng-Wei Hsieh, Hsu-Chieh Chang, Yi-Hao Chen, Ke-Hung Chien

**Affiliations:** ^1^Department of Ophthalmology, Taoyuan Armed Forces General Hospital, Taoyuan, Taiwan; ^2^National Defense Medical Center, Taipei, Taiwan; ^3^Department of Nursing, Tri-Service General Hospital and National Defense Medical Center, Taipei, Taiwan; ^4^Graduate Institute of Nursing, College of Nursing, Taipei Medical University, Taipei, Taiwan; ^5^Department of Ophthalmology, Tri-Service General Hospital, Taipei, Taiwan

**Keywords:** myopia, atropine, low-dose atropine, pediatric ophthalmology, myopic control

## Abstract

**Purpose:**

Myopia is a disorder of growing prevalence in school-aged children worldwide, especially in Asia. Although low-dose atropine is recognized as an effective treatment to slow myopia progression, different studies have reported varying efficacies of treatment, and the optimal concentration of low-dose atropine remains an open question.

**Methods:**

A two-stage approach was conducted in this study. First, an observational study was conducted to plot the axial length growth curve for Taiwanese children. Second, an interventional 2-year study was performed in which different concentrations of low-dose atropine were applied based upon the risk-level status from the first stage.

**Results:**

A total of 4,091 subjects, consisting of 2,105 boys (51.5%) and 1,986 girls (48.5%), were enrolled in the first stage to plot the axial growth curve for Taiwanese children aged between 3 and 16 years. The percentage of children with myopia increased from 2.3% in 4-year-olds to 88.0% in 16-year-olds. At the second stage, a total of 886 subjects [307 (34.65%) at low risk, 358 (40.41%) at moderate risk and 221 (24.94%) at high risk] were enrolled to receive low-dose atropine based upon the risk level (0.02, 0.03, and 0.05%, respectively). With this approach, the mean annual myopia progression was −0.33, −0.57, and −0.82 D in the low-risk, moderate-risk and high-risk groups, respectively. Applying annual myopic progression < -1.0 D as a criterion for responder, the responder rates were 95.77, 83.52, and 70.59% in the low-risk, moderate-risk, and high-risk groups, respectively.

**Conclusions:**

We proposed a classification-based approach involving different concentrations of low-dose atropine based upon an individual's risk-level status. With this approach, myopic progression can be effectively controlled in patients without exposure to atropine side effects due to exposure to a higher dose than actually needed.

## Introduction

Myopia is one of the most prevalent diseases around the world, especially in Asia (1). It is recognized as a multifactorial disorder associated with different geographic areas, races, ages, and environmental causes (1). Nonetheless, its prevalence continues to increase worldwide. In the most rapidly growing areas, such as Taiwan in Asia, the prevalence of myopia has grown from 5.8% in 1983 to 21% in 2000 and 25.41% in 2017 among 7-year-old children (2, 3). Early onset of myopia has been recognized as a risk factor for high myopia in adulthood (4). The prevalence of high myopia in 15-year-olds has also grown from 4.37% in 1983 to 15.36% in 2017 (3). Due to the increasing time spent in near work or electric devices, which are recognized as one of its major risk factors, myopia control remains an unsolved problem.

Atropine, a non-specific muscarinic inhibitor, is currently the main medication to effectively control myopic progression (5, 6). This compound has been shown to demonstrate a better myopia control effect at proportionally higher doses, but side effects such as photophobia, allergic conjunctivitis and accommodation insufficiency are also increased (7, 8). Low-dose atropine (0.01%) has received high interest given its non-inferior efficacy but fewer side effects following publication of the ATOM2 study. Recently, different studies have also showed better control of myopia progression with concentrations of low-dose atropine higher than 0.01%, exceeding those of traditional high-dose atropine, with acceptable side effects (9–11). Since the efficacy of low-dose atropine varies among different studies in different concentrations and races, there is still no optimal concentration for treating myopic individuals.

In this study, we proposed a categorized approach for delivering different concentrations of atropine in myopic children according to their risk classification based upon axial length (AL) percentiles. Estimated annual spherical equivalent (SE) progression and AL growth were measured and evaluated to adjust the atropine concentration at subsequent visits. The average annual SE progression during the 2-year study period was calculated to evaluate the efficacy of this method.

## Methods

The current prospective study was conducted on patients who underwent myopia control at Tri-Service General Hospital between January 2015 and December 2019. The protocol and related documents were reviewed and approved (No: 2-104-05-031) by the Institutional Review Board of the Tri-Service General Hospital, Taipei, Taiwan. Informed consent was reviewed by the review board, and the study was performed under the Good Clinical Practice guidelines of Taiwan and the Declaration of Helsinki, 1964. All children who fulfilled the below mentioned criteria of the study were invited to participate, and informed consent was signed by the children and the parents.

**Figure 1:**
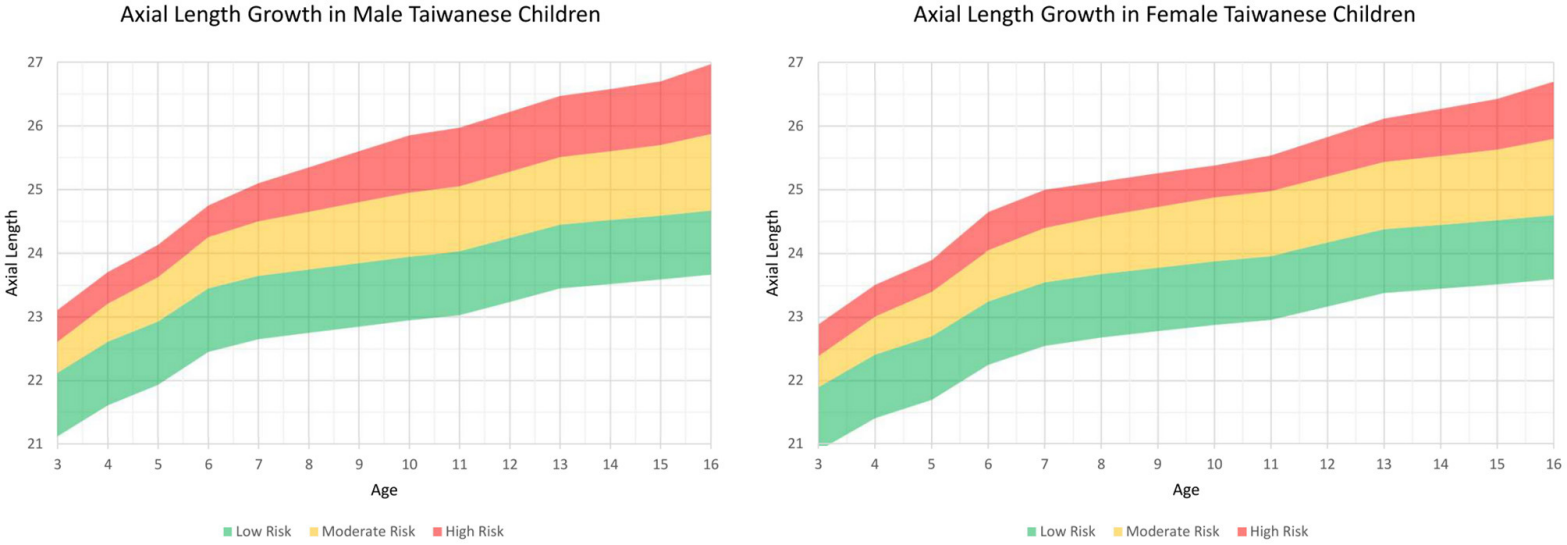
Axial length growth chart among Taiwanese children separated by sex from the first part of this study. The charts were further subclassified the percentiles into three risk categories: below the 50th percentile, low risk (green color); 50th to 90th percentile, moderate risk (yellow color); and above the 90th percentile, high risk (red color).

The current study was divided into two parts:

The first part validated the growth of axial length in Taiwanese myopes (Figure 1). Patients were eligible for inclusion in this part of the study if they had been to the vision evaluation clinic in the Tri-Service General Hospital, were between 3 and 16 years old and had no comorbidity of other ocular diseases that could result in reduced vision, such as amblyopia, congenital glaucoma, pediatric cataracts, and trauma-related events. Patients were excluded from the study if they had other concurrent ocular diseases that might result in reduced vision or if they could not complete the full evaluations in the clinic.

The goal of the second part of the study was to evaluate the efficacy of stepwise control in myopia progression via biaxial approaches. Patients were enrolled in this part of the study if they were aged 4–16 years old, had been diagnosed with myopia [spherical equivalent (SE) < -0.5 D] and not been treated with atropine or other myopic treatments, followed up in a myopic clinic for at least 2 years and were aged between 4 and 18 years old during the follow-up periods. Patients were excluded if they had not been diagnosed with myopia and had other diseases that could hinder their vision, such as amblyopia and pediatric glaucoma. A follow-up period of <2 years was also a criterion for exclusion.

**Figure 2:**
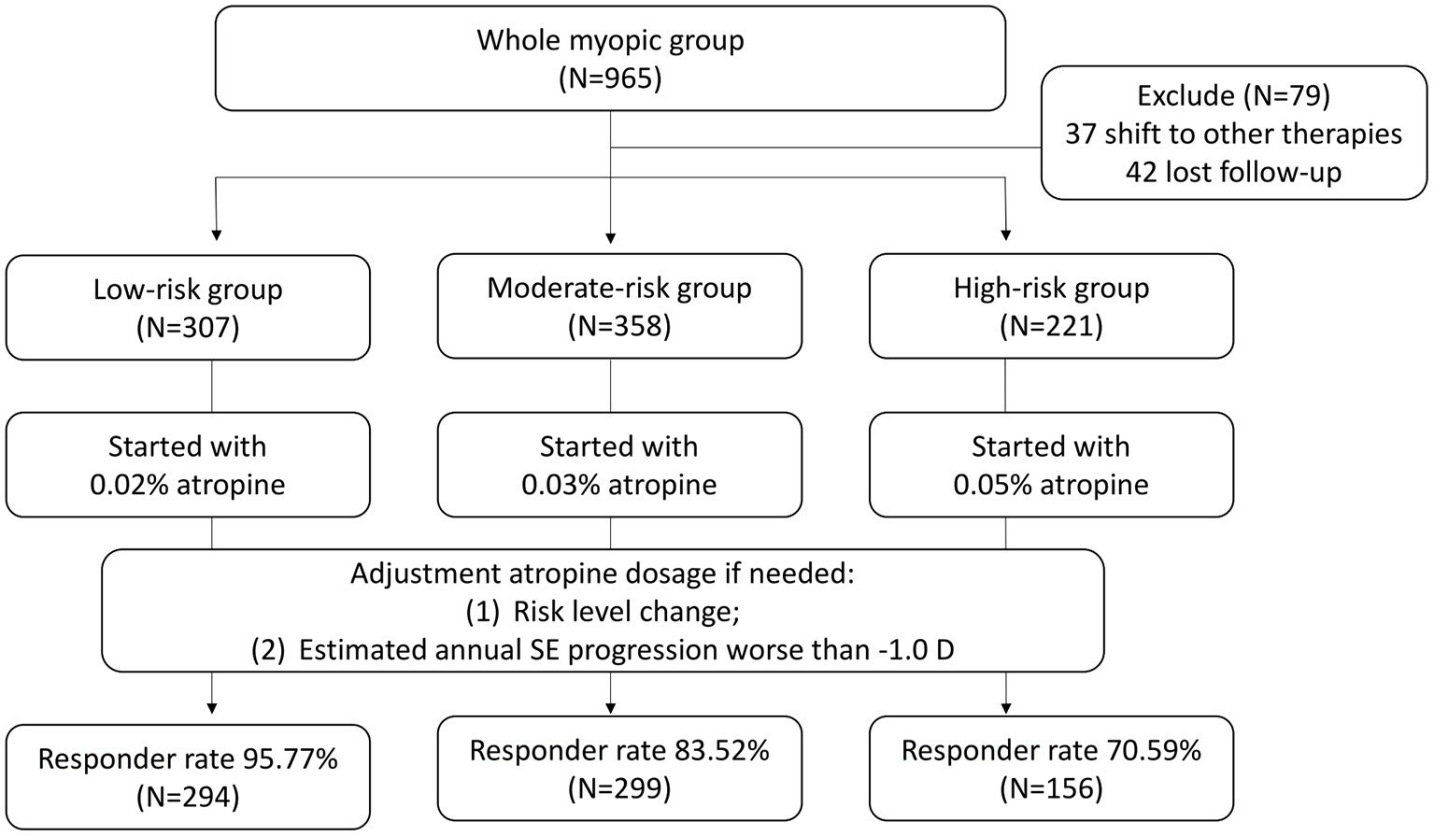
The study protocol of the second part of the study. The atropine concentration for starting treatment was individualized based on the patient's risk classification from the growth chart derived from the first part of this study from 0.02, 0.03, and 0.05%, respectively. SE, spherical equivalent.

The stepwise control is based on that from Wu et al. with a threshold for increasing atropine concentration by myopia progression of more than −0.5 D within 6 months (12, 13). However, the atropine concentration for starting treatment was individualized based on the patient's risk classification from the growth chart derived from the first part of this study. The study protocol is plotted in Figure 2. Patients whose axial length was located below the 50th percentile (low risk, green color) were given 0.02% atropine as a starting medication. Those whose axial length was located between the 50th and 90th percentiles (moderate risk, yellow color) were given 0.03% atropine, and those whose axial length was located above the 90th percentile (high risk, red color) was given 0.05% atropine. At the first visit and the follow-up visits every 3 months during the 2-year follow-up period, patients were evaluated in terms of the best-corrected visual acuity (BCVA), refraction in SE, and AL. During the follow-up visits, patients were evaluated for medication adjustment according to two simple parameters. One was the risk status change, and the other was the estimated annual SE progression. If the two parameters indicated better status (the risk status dropped to a safer status (e.g., moderate risk to low risk or high risk to moderate risk) and the estimated annual SE progression was better than −1.0 D), the atropine dose was decreased (e.g., 0.05–0.03% atropine). If the two parameters indicated worse status [the risk status increased to a higher status (e.g., moderate risk to high risk or low risk to moderate risk) and the estimated annual SE progression was worse than −1.0 D], the atropine dose was increased (e.g., 0.03–0.05% atropine). If the two parameters indicated status stability (the risk status stayed the same and the estimated annual SE progression was better than −1.0 D), the atropine dose was unchanged. If the two parameters indicated an uncertain status change, such as worse risk status change but good control of the estimated annual SE progression and better or stable risk status with poor control of the estimated annual SE progression, the decision to adjust the atropine dose was left to the physician. The change of low atropine regimen was unlimited to times and concentration during the following up period. During the study, full correction of refraction error was prescribed for all the participants.

The following parameters were collected from the charts for the study: age, sex, best-corrected visual acuity, refraction in SE, AL, and medications at each visit. AL was measured by a non-invasive and non-contact biometer, an AL-Scan (Nidek Co., Ltd., Aichi, Japan). Measurement of SE was performed by a TONOREF III autorefractor (Nidek Co., Ltd., Aichi, Japan) and was obtained under full cycloplegic status (instillation of 4 drops of Mydrin-P (Santen Pharmaceutical Co., Ltd. Shiga, Japan) at 5-min intervals and observation to check non-reactivity to light 30 min after the last drop). Only the data from the right eye were recorded for analysis.

Outcome measures were recorded from the charts as a criterion of responder: average annual myopic progression < -1.0 D at the end of the 2-year study.

The data were analyzed using SPSS software version 18.0 for Windows (SPSS Inc., Chicago, IL, USA). Non-normal distribution was confirmed with the kolmogorov-smirnov normality test. And then non-parametric statistics were applied in the study. The Chi-Square Test was applied in the difference of categorical variables between two groups while the Fisher Exact Test was applied in certain variables. The Mann-Whitney *U* test was applied in the difference of continuous variables between two groups. A *P* < 0.05 was considered statistically significant.

## Results

In the first part of the study, 4,091 Taiwanese children were enrolled, with 2,105 boys (51.5%) and 1,986 girls (48.5%). There was a mean SE of −0.58 D (SD 2.47) with minimum SE −7.5D and maximum SE +10.5D among the participants. Detailed information on the prevalence of myopia and AL (mean ±*SD*), separated by age and sex, is listed in Table 1. Among first graders (7 years old), there was a noticeable percentage of myopia in both sexes (40.3% for boys and 37.8% for girls). The prevalence of myopia increased with age and was particularly prominent among school-aged children. Among third graders (9 years old), more than half had myopia (56.4% for boys and 53.8% for girls), while among sixth graders (12 years old), the percentage of myopia was over 80% in both sexes (82.4% for boys and 83.2% for girls). In our cohort, the increasing incidence of myopia with age stabilized by 13 years of age, but a relatively high percentage of myopia remained in both sexes (Table 1).

**Table 1:** Demographic characteristics of children in the first part of the study.

**Age (y/o)**	**Male subjects**			**Female subjects**			**Whole cohort**	
	**AL (mm)**	**Myopic percentage (%)**	**Cases (*N*)**	**AL (mm)**	**Myopic percentage (%)**	**Cases (*N*)**	**Myopic percentage (%)**	**Cases (*N*)**
3	22.12	0.0	112	21.90	0.0	141	0.0	253
4	22.61	3.3	190	22.41	1.2	154	2.4	344
5	22.93	4.7	268	22.70	3.6	309	4.1	577
6	23.42	15.2	145	23.25	12.3	257	13.3	402
7	23.63	40.3	201	23.56	37.8	154	39.2	355
8	23.76	47.6	268	23.67	46.2	116	47.2	384
9	23.85	56.4	179	23.80	53.8	180	55.1	359
10	23.94	70.2	156	23.87	67.3	129	68.9	285
11	24.03	81.8	179	23.98	79.5	167	80.7	346
12	24.24	82.4	268	24.15	83.2	141	82.7	409
13	24.45	85.9	67	24.39	86.6	141	86.4	208
14	24.52	89.4	34	24.46	87.3	64	88.0	98
15	24.59	90.4	28	24.55	88.2	19	89.5	47
16	24.67	88.5	11	24.64	87.6	13	88.0	24

*AL, Axial Length*.

Information on the AL measurements separated by age and sex is also listed in Table 1. A sex difference was apparent and tended to decrease with age. In addition, AL also increased with age, and the annual AL growth was faster before 7 years of age and between 11 and 13 years of age in both sex (Table 1). Using the AL measurements from the children in the first stage, we plotted the axial length growth chart among Taiwanese children and sub-classified the percentiles into three risk categories: below the 50th percentile, low risk (green color); 50 to 90th percentile, moderate risk (yellow color); and above the 90th percentile, high risk (red color) (Figure 1).

In the second part of the study, 965 patients were initially invited to participate in the study. At the first visit, they were classified into different risk statuses based on the growth chart plotted using the initial AL measurements at the first visit and given different starting atropine doses according to their risk status. During the 2-year follow-up period, patients were asked to return every 3 months and evaluate the need for medication adjustment. During the study period, 37 patients shifted to other therapies (31 with orthokeratology and 6 with myopia-controlled glasses), and 42 could not complete the 2-year follow-up requirement. In the end, 886 patients (439 boys and 447 girls) fulfilled the study criteria, and their information was collected for analysis. All the patients enrolled in the study followed the treatment protocol for at least 2 years. At the end of the study, 307 patients (171 male and 136 female) were in the low-risk status at baseline, 358 in the moderate-risk status (156 male and 202 female) and 221 in the high-risk status (112 male and 109 female). Their data was collected for further analysis (Table 2). Information and study results of subjects stratified by age and risk level was listed in Supplementary Tables 1, 2.

**Table 2:** Demographic characteristics of enrolled children in the second part of the study.

	**Low-risk group**	**Moderate-risk group**	**High-risk group**	**Whole group**	***p***
	**(*N* = 307)**	**(*N* = 358)**	**(*N* = 221)**	**(*N* = 886)**	
**Sex**					
Male (*N*) (%)	171 (55.7%)	156 (43.6%)	112 (50.7%)	439 (49.5%)	0.12
Female (*N*) (%)	136 (44.3%)	202 (56.4%)	109 (49.3%)	447 (50.5%)	0.15
Baseline Age (year old) (mean) (*SD*)	9.98 (2.65)	9.45 (2.99)	9.56 (3.43)	9.66 (3.92)	0.21
Baseline SE (D) (mean) (*SD*)	−0.84 (0.57)	−1.36 (0.63)	−2.49 (0.75)	−1.46 (0.62)	<0.01^*^
Baseline AL (mm) (mean) (*SD*)	23.39 (3.20)	24.23 (2.98)	25.02 (3.14)	24.14 (3.05)	<0.01^*^

P value in the table is from values compared among different risk level groups. SE, spherical equivalent; AL, axial length; N, number; D, diopter; SD, standard deviation. ^*^P < 0.05, significance.

The mean annual SE progression of the whole group was −0.46 D (SD 0.23), with a mean AL growth of 0.36 mm (*SD* 0.26). There were 379 male patients (82.6%) and 370 female patients (86.7%) who fulfilled the criterion of responder at the end of the 2-year study (Table 2). In the subgroup analysis, patients in the low-risk group at the beginning revealed the highest responder rate (294, 95.77%) among both male (171 boys) and female (136 girls) patients. In this subgroup, the mean myopic progression was −0.35 D (*SD* 0.23) in male patients and −0.31 D (*SD* 0.27) in female patients (*P* = 0.53). The mean AL growth was 0.14 mm (*SD* 0.09) in male patients and 0.12 mm (*SD* 0.08) in female patients (*P* = 0.47). In the moderate-risk subgroup, 127 out of 156 male patients (81.4%) and 159 out of 202 female patients (78.7%) met the responder criteria (*P* = 0.15). The mean myopic progression was −0.51 D (SD 0.32) in male patients and −0.62 D (*SD* 0.47) in female patients (*P* = 0.53). The mean AL growth was 0.33 mm (*SD* 0.21) in male patients and 0.35 mm (*SD* 0.23) in female patients (*P* = 0.27). In the high-risk subgroup, there was a lower responder rate in both male (81, 72.3%) and female patients (75, 68.7%; *P* = 0.13). The mean myopic progression in the high-risk subgroup was −0.73 D (*SD* 0.36) in male patients and −0.91 D (*SD* 0.62) in female patients (*P* = 0.09). The mean AL growth was 0.73 mm (*SD* 0.12) in male patients and 0.57 mm (*SD* 0.22) in female patients (*P* = 0.12) (Table 3).

**Table 3:** Study results of enrolled children in the second part of the study.

	**Low-risk group**	**Moderate-risk group**	**High-risk group**	**Whole group**	***p***
	**(*N* = 307)**	**(*N* = 358)**	**(*N* = 221)**	**(*N* = 886)**	
Annual SE progression (D) (mean) (*SD*)	−0.33 (0.30)	−0.57 (0.39)	−0.82 (0.44)	−0.55 (0.36)	<0.01^*^
Male (D) (mean) (*SD*)	−0.35 (0.23)	−0.51 (0.32)	−0.73 (0.36)	−0.50 (0.31)	<0.01^*^
Female (D) (mean) (*SD*)	−0.31 (0.27)	−0.62 (0.47)	−0.91 (0.62)	−0.60 (0.41)	<0.01^*^
Annual AL growth (mm) (mean) (*SD*)	0.13 (0.08)	0.34 (0.20)	0.65 (0.36)	0.35 (0.21)	<0.01^*^
Male (mm) (mean) (*SD*)	0.14 (0.09)	0.33 (0.21)	0.73 (0.49)	0.36 (0.20)	<0.01^*^
Female (mm) (mean) (*SD*)	0.12 (0.08)	0.35 (0.18)	0.57 (0.22)	0.33 (0.17)	<0.01^*^
Responder (*N*) (%)	294 (95.77%)	299 (83.52%)	156 (70.59%)	749 (84.54%)	<0.01^*^
Male (*N*) (%)	163 (95.3%)	135 (86.5%)	81 (72.3%)	379 (82.6%)	<0.01^*^
Female (*N*) (%)	131 (96.3%)	164 (81.2%)	75 (68.7%)	370 (86.7%)	<0.01^*^

*P value in the table is from values compared among different risk level groups. N, number; D, diopter; SD, standard deviation*.

^*^P < 0.05, significance.

Under the study protocol, subjects were allowed to change their low atropine concentration in 3-month follow-up visits by two evaluation criteria: (1) myopic risk status change; (2) the estimated annual SE progression. The change of low atropine concentration was unlimited to times and concentration during the following visits. As a result, there was a mean 9.45% (29 subjects, 13 male and 16 female) of low-risk level subjects who had been received regimen change during the study period. As a comparison, 25.98% subjects (45 male and 48 female) in the moderate-risk level and 32.13% subjects (37 male and 34 female) in the high-risk level underwent dosage change during the study period. Detailed information about SE and AL progression before and after atropine concentration change was listed in Supplementary Table 3.

Photophobia was reported during the initial 2 weeks by 13 patients (4.2%) with the low-risk level, 18 (5.0%) with the moderate-risk level and 14 (6.3%) with the high-risk level (*P* = 0.31). In the following visits, the symptoms of photophobia improved in each subject, and none of the mentioned patients dropped out of the study or discontinued medication due to photophobia. In addition to photophobia, 2 patients (0.7%) at the low-risk level, 7 (2.0%) at the moderate-risk level and 5 (2.3%) at the high-risk level experienced allergic conjunctivitis (*P* = 0.12). All cases were temporary and mild after medication.

## Discussion

In the management of pediatric myopia, low-dose atropine is approved with good efficacy at different doses (9, 10, 14). However, there is no ideal dose for different individuals. Our study proposed a classification approach involving different doses of atropine according to risk categories from AL percentiles. We started with 0.02, 0.03, and 0.05% atropine in low-risk, moderate-risk, and high-risk groups, respectively. It is known that atropine has a significant dose-dependent effect on refractive change, axial elongation, and adverse effects (15). We choose 0.02% atropine as a starting dose instead of 0.01% in low-risk group based upon below reasons: (1) 0.02% atropine had a better effect on myopia progression than 0.01% atropine, but 0.02% and 0.01% atropine showed similar effects on pupil diameter and accommodative amplitude (16). (2) Our prior study showed 0.01% atropine has a 58.63% responder rate in Taiwan myopic children but has better effect in certain children whose myopia better than −1.5D and age younger than 9 years when starting atropine treatment (17). About a choice of 0.05% atropine as a starting concentration in high risk group was due to that pupil dilation by 0.05% atropine is still tolerable in children between 4 and 12 years old (18). Besides, up regulation of atropine dosage was agreed in this trial based upon adjustment criteria in study protocol. And choosing 0.03% atropine in moderate-risk group is from mid-range between 0.02 and 0.05% concentration under the dose-dependent characteristics of atropine. This classified approach demonstrated a high responder rate of 84.54% in a 2-year follow-up.

In 2017, the prevalence of myopia in 7-year-olds in Taiwan reached up to 25.41% (3). To better control the prevalence of progressing myopia, a government-supported welfare program was instituted for school-aged children in Taiwan in 2013 that provides annual eye examinations by an ophthalmologist to first-grade children (~6–7 years old) without extra cost. Although the prevalence of myopia has continued to increase since release of the report (3), this was the least-biased prevalence relative to prior studies in Taiwan.

From the perspective of different initial risk levels, children at high-risk levels demonstrated a lower responder rate than children at low and moderate-risk levels. The 70.59% (72.3% in males and 68.7% in females) responder rate at the high-risk level in the current study is lower than the 84.8~93.2% responder rate with 0.05% atropine reported in other studies (9, 10, 19). Owing to the composition of the study population, it is reasonable that our study demonstrated a lower responder rate in high-risk level patients. The lower responder rate and higher annual myopia progression (−0.82 D) implied that in these patients, there was a higher proportion of children who may need higher dose of atropine or other alternative treatments to effectively control their myopic progression.

Race is also a prominent factor in the differences in myopic control. In European studies, 0.01% atropine had good efficacy in limiting the annual myopia progression by ~-0.4 to −0.5 D (20, 21). However, a higher concentration of low-dose atropine is needed in Asian patients to provide similar effects. In LAMP studies with Chinese subjects, annual myopia progression was observed in the 0.025% atropine-treated group (9, 10). In another study involving Korean subjects, even 0.025% atropine could only achieve −0.56 D annual myopia progression (19). In the current study, we applied low-dose atropine in Taiwanese subjects of Han ethnicity in Southeast Asia with a similar effectiveness −0.57D in the group of 0.03% atropine. Although there is no related reference for low-dose atropine in general myopic children, moderate-risk subjects who were treated with 0.03% atropine at the beginning of the study demonstrated an annual myopia progression of −0.57 D. With different concentrations of low-dose atropine for classifying risk level in the current study, we achieved a mean annual myopia progression of −0.46 D in our population. In patients at the high-risk level, even 0.05% atropine could only provide fair myopia control at −0.82 D annual myopic progression, comparable to control groups in certain studies (9, 20, 22). This implies that higher concentrations of atropine or other alternatives may be needed in these patients. From our study, we believe that the identification of high-risk level patients before myopic treatment is better for long-term myopic control.

Younger age at myopia onset is associated with a higher risk of high myopia in adulthood (23). In another recent study, younger age was a risk factor for poor response to low-dose atropine, and the authors suggested that 0.05% atropine should be used in younger children for better myopia control (14). Li et al. stated that age is the only factor associated with atropine response in myopia control, not baseline SE or parental myopia status (14). Wu et al. suggested increasing the concentration of low-dose atropine from 0.01 (13) or 0.05% (24) in a stepwise manner with a clinical judgment of myopia progression of 0.5 D over a 6-month period. However, 0.05% atropine is associated with photophobia side effects in some children, and it is a concern in clinical practice (25). Although a higher concentration is associated with a higher response in myopia control, we classified our subjects based upon risk level and prescribed different concentrations of low-dose atropine. In subjects of all ages in the low-risk group (ranging from 2.98% at 4 years old to 46.61% at 15 years old), our approach demonstrated 95.77% responder in myopia control with 0.02% atropine. However, in the high-risk group (ranging from 3.77% in 15-year-old to 30.51% in 4-year-old), subjects achieved an overall acceptable 70.59% responder rate with a starting atropine concentration of 0.05%. This implies that with adequate low-dose atropine, certain children, especially those at low-risk levels, could have good myopia control in any age group.

The efficacy of low-dose atropine in myopic control varies among different studies with different designs and baseline subject characteristics. In the ATOM2 phase 1 study in subjects aged between 6 and 12 years with baseline myopia of at least −2.0 D, subjects under 0.01% atropine demonstrated −0.43 D and −0.49 D myopic progression in the first and second years, respectively (8). In another study conducted in children aged 5–14 years with baseline myopia of under −6.0 D, subjects demonstrated an annual myopia progression of −0.84, −0.56, and −0.23 D in the 0.01, 0.025, and 0.05% atropine-treated groups, respectively (19). In the LAMP1 study in children aged between 4 and 12 years with at least −1.0 D myopia, the subjects demonstrated an annual myopia progression of −0.59, −0.46, and −0.27 D with 0.01, 0.025, and 0.05% atropine control, respectively (9). In our study, different concentrations of low-dose atropine were applied to children with different risk levels of myopia; 84.54% (749 over 886 patients) reached the responder criteria by the end of the study and had a mean −0.46 D in annual myopia progression, which is comparable to previous studies on single low-dose atropine. From the composition of the study subjects, which consisted of 34.65% low-risk subjects (307 patients), 40.41% moderate-risk subjects (358 patients) and 24.94% high-risk subjects (221 patients), we believed that this approach could have similar efficacy without unnecessary side effects and could be referenced by ophthalmologists in real-world practice.

There were still some limitations in this study. First, there was no control group in this study and the part 1 study result was limited due to a hospital-based population study instead of school screening. We adopted normal AL growth in the first part of the study and administered low-dose atropine in the second part of the study. Since this was not a randomized study, but an open study based upon myopia risk level, there was no way to evaluate a control group at each risk level. Further study may be needed to address this matter. Second, 79 patients (8.2%) dropped out from the second part of the study. Since our study limited physicians to shifting medications among different, low doses of atropine, some patients may have shifted to other treatment choices, such as orthokeratology or myopia control spectacles. Further studies addressing wider treatment choices are needed to evaluate their efficacy. Third, our study only provided 2-year study results. Midterm results covering 3–5 years are under evaluation and will be reported in the future.

In conclusion, we conducted a two-stage approach to control myopia progression with different concentrations of low-dose atropine based upon risk classification. With this individualized approach, we achieved a non-inferior result compared to other studies applying a single concentration of low-dose atropine in generally myopic children.

We believe our study results could serve as a reference for ophthalmologists to initiate studies of low-dose atropine treatment guideline in myopic children.

## Data Availability Statement

The raw data supporting the conclusions of this article will be made available by the authors, without undue reservation.

## Ethics Statement

The studies involving human participants were reviewed and approved by the Institutional Review Board of the Tri-Service General Hospital, Taipei, Taiwan. Written informed consent to participate in this study was provided by the participants' legal guardian/next of kin.

## Author Contributions

M-WH design of the study, conduct of the study, data collection, and data analysis. M-WH and K-HC interpretation of the data. K-HC preparation and review. K-HC and Y-HC approval of the manuscript. All authors contributed to the article and approved the submitted version.

## Funding

This research was funded by the Tri-Service General Hospital (TSGH) (TSGH-D-110115 and TSGH-D-110111), the Taoyuan Armed Forces General Hospital (TYAFGH-E-111044 and TYAFGH-A-110019), the Taiwan Ministry of Science and Technology (MOST 110-2314-B-016-051), and the Ministry of National Defense Medical Affairs Bureau (MND-MAB-C05-111019).

## Conflict of Interest

The authors declare that the research was conducted in the absence of any commercial or financial relationships that could be construed as a potential conflict of interest.

## Publisher's Note

All claims expressed in this article are solely those of the authors and do not necessarily represent those of their affiliated organizations, or those of the publisher, the editors and the reviewers. Any product that may be evaluated in this article, or claim that may be made by its manufacturer, is not guaranteed or endorsed by the publisher.
