# Supplementary material for: Classification-Based Approaches to Myopia Control in a Taiwanese Cohort
Source: Front Med (Lausanne). 2022 Jun 10;9:879210. doi: 10.3389/fmed.2022.879210 (PMC9226386; doi:10.3389/fmed.2022.879210)
Supplement: Supplementary file 1 [file Table_1.docx]

| **Supplementary Table 1.** Subjects stratified by age and risk-level grouping in the second part of the study | | | | | | | | | | | |
| --- | --- | --- | --- | --- | --- | --- | --- | --- | --- | --- | --- |
|  | Male Subjects (N) (%) | | | | Female Subjects (N) (%) | | | | Whole Cohort | | |
| Age (y/o) | | Low-risk | Moderate-risk | High-risk | | Low-risk | Moderate-risk | High-risk | | Subjects (N) | Percentage (%) |
| 4 | | 1 (0.58) | 6 (3.85) | 4 (3.57) | | 0 (0) | 10 (4.95) | 3 (2.75) | | 24 | 2.71 |
| 5 | | 2 (1.17) | 9 (5.77) | 12 (10.71) | | 1 (0.74) | 15 (7.43) | 9 (8.26) | | 48 | 5.42 |
| 6 | | 17 (9.94) | 12 (7.69) | 9 (8.04) | | 19 (13.97) | 21 (10.40) | 11 (10.09) | | 89 | 10.05 |
| 7 | | 22 (12.87) | 13 (8.33) | 11 (9.82) | | 17 (12.50) | 23 (11.39) | 8 (7.34) | | 94 | 10.61 |
| 8 | | 21 (12.28) | 20 (12.82) | 13 (11.61) | | 15 (11.03) | 24 (11.88) | 14 (12.84) | | 107 | 12.08 |
| 9 | | 18 (10.53) | 20 (12.82) | 11 (9.82) | | 14 (10.29) | 19 (9.41) | 8 (7.34) | | 90 | 10.16 |
| 10 | | 17 (9.94) | 19 (12.18) | 11 (9.82) | | 14 (10.29) | 18 (8.91) | 13 (11.93) | | 92 | 10.38 |
| 11 | | 13 (7.60) | 14 (8.97) | 8 (7.14) | | 15 (11.03) | 19 (9.41) | 10 (9.17) | | 79 | 8.92 |
| 12 | | 21 (12.28) | 11 (7.05) | 10 (8.93) | | 15 (11.03) | 14 (6.93) | 7 (6.42) | | 78 | 8.80 |
| 13 | | 15 (8.77) | 10 (6.41) | 8 (7.14) | | 9 (6.62) | 11 (5.45) | 6 (5.50) | | 59 | 6.66 |
| 14 | | 9 (5.26) | 10 (6.41) | 7 (6.25) | | 3 (2.21) | 12 (5.94) | 11 (10.09) | | 52 | 5.87 |
| 15 | | 8 (4.68) | 7 (4.49) | 5 (4.46) | | 5 (3.68) | 9 (4.46) | 4 (3.67) | | 38 | 4.29 |
| 16 | | 7 (4.09) | 5 (3.21) | 3 (2.68) | | 9 (6.62) | 7 (3.47) | 5 (4.59) | | 36 | 4.06 |
| Total | | 171 | 156 | 112 | | 136 | 202 | 109 | | 886 | 100 |
|  | | | | | | | | | | | |
