# Supplementary material for: Classification-Based Approaches to Myopia Control in a Taiwanese Cohort
Source: Front Med (Lausanne). 2022 Jun 10;9:879210. doi: 10.3389/fmed.2022.879210 (PMC9226386; doi:10.3389/fmed.2022.879210)
Supplement: Supplementary file 2 [file Table_2.docx]

| **Supplementary Table 2.** Study results of enrolled children stratified by age in the second part of the study | | | | |  |
| --- | --- | --- | --- | --- | --- |
|  | Low-risk group  (N=307) | Moderate-risk group  (N=358) | High-risk group  (N=221) | P | |
| Age 4-6 years-old |  |  |  |  | |
| Subjects (N) (%) | 40 (13.03) | 73 (20.39) | 48 (21.72) | 0.12 | |
| Annual SE progression (D) (mean) (SD) | -0.30 (0.19) | -0.51 (0.22) | -0.60 (0.26) | <0.01* | |
| Annual AL progression (mm) (mean) (SD) | 0.19 (0.09) | 0.31 (0.10) | 0.54 (0.12) | <0.01* | |
| Responder (N) (%) | 39 (97.50) | 66 (90.41) | 39 (81.25) | <0.01* | |
| Age 7-12 years-old |  |  |  |  | |
| Subjects (N) (%) | 202 (65.80) | 214 (59.78) | 124 (56.11) | 0.13 | |
| Annual SE progression (D) (mean) (SD) | -0.35 (0.16) | -0.59 (0.31) | -0.92 (0.38) | <0.01* | |
| Annual AL progression (mm) (mean) (SD) | 0.13 (0.07) | 0.37 (0.14) | 0.70 (0.17) | <0.01* | |
| Responder (N) (%) | 194 (96.04) | 174 (81.31) | 89 (71.77) | <0.01* | |
| Age 13-16 years-old |  |  |  |  | |
| Subjects (N) (%) | 65 (21.17) | 71 (19.83) | 49 (22.17) | 0.23 | |
| Annual SE progression (D) (mean) (SD) | -0.29 (0.15) | -0.55 (0.26) | -0.76 (0.28) | <0.01* | |
| Annual AL progression (mm) (mean) (SD) | 0.13 (0.06) | 0.34 (0.11) | 0.65 (0.19) | <0.01* | |
| Responder (N) (%) | 61 (93.85) | 59 (83.10) | 28 (57.14) | <0.01* | |
| P value in the table is from values compared among different risk level groups. SE = spherical equivalent, AL = axial length, N = number, D = diopter; SD = standard deviation. *P<0.05 = significance. | | | | | |
