# Supplementary material for: Classification-Based Approaches to Myopia Control in a Taiwanese Cohort
Source: Front Med (Lausanne). 2022 Jun 10;9:879210. doi: 10.3389/fmed.2022.879210 (PMC9226386; doi:10.3389/fmed.2022.879210)
Supplement: Supplementary file 3 [file Table_3.docx]

| **Supplementary Table 3.** Parameters of subjects before and after atropine concentration change in the second part of study | | | | |  |
| --- | --- | --- | --- | --- | --- |
|  | Low-risk group  (N=29) | Moderate-risk group  (N=93) | High-risk group  (N=71) | Whole group  (N=193) | |
| Sex  Male (N)  Female (N) | 13  16 | 45  48 | 37  34 | 95  98 | |
| Before atropine concentration change  Estimated annual SE progression (D) (mean) (SD)  Estimated annual AL growth (mm) (mean) (SD)  After last atropine concentration change* | -1.27 (0.55)  0.28 (0.11) | -1.46 (0.61)  0.46 (0.09) | -1.53 (0.47)  0.66 (0.13) | -1.46 (0.51)  0.51 (0.11) | |
| Estimated annual SE progression (D) (mean) (SD) | -0.83 (0.21) | -0.75 (0.24) | -0.92 (0.19) | -0.82 (0.20) | |
| Estimated annual AL growth (mm) (mean) (SD) | 0.19 (0.10) | 0.29 (0.08) | 0.48 (0.12) | 0.34 (0.09) | |
| SE = spherical equivalent, AL = axial length, N = number, D = diopter, SD = standard deviation. *Atropine concentration change was not limited to once in the study and the information of estimated annual change was presented from last change in the study period. | | | | | |
